# Research on Longitudinal and Lateral Game Control of Intelligent Agricultural Machine Stackelberg Based on Takagi‐Sugeno Fuzzy Model

**DOI:** 10.1002/fsn3.70579

**Published:** 2025-09-10

**Authors:** Guangfei Xu, Dexi Wu, Haizhu Xu, Hequan Miao, Yulong Chen

**Affiliations:** ^1^ School of Mechanical and Automotive Engineering Liaocheng University Liaocheng China; ^2^ Shandong Polytechnic Technician College Liaocheng China; ^3^ School of Agricultural Engineering and Food Science Shandong University of Technology Zibo China

**Keywords:** information interaction, longitudinal and lateral control, parameter variation, T‐S Stackelberg

## Abstract

Aiming at the coupling interference problem of intelligent driving agricultural machinery in the longitudinal and lateral control, and the real‐time change of key parameters in the process of establishing the dynamic model, which seriously reduces the control performance of the controller, etc. In this paper, a Takagi‐Sugeno (T‐S) Fuzzy Model Stackelberg game control algorithm (T‐S Stackelberg game control) is proposed. Firstly, according to the characteristics of the model, the change parameters in the model were determined. Secondly, the T‐S fuzzy model was designed, and the corresponding membership functions were determined. Thirdly, the T‐S Stackelberg game controller was designed. Finally, the reliability of the proposed algorithm is verified by a hardware‐in‐the‐loop experiment.

## Graphical abstract

This paper proposes a Stackelberg game‐based fuzzy control algorithm with T‐S modeling for autonomous agricultural machinery. This framework resolves lateral‐longitudinal control conflicts through hierarchical game‐theoretic interactions between control layers. By embedding optimization objectives within adaptive fuzzy rule antecedents, it achieves computationally efficient multi‐objective decision‐making in real‐time field operations. The figure that best represents the scope of the paper.

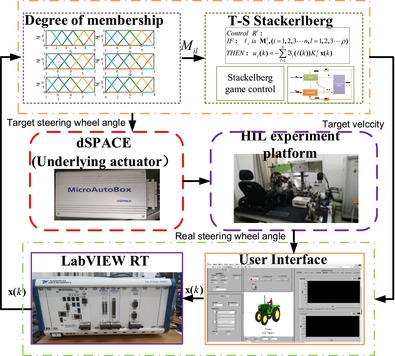

## 1 Introduction

With the rapid development of intelligent technology, the agricultural field is experiencing an unprecedented change. As an important and indispensable machine in agricultural production, the intelligent upgrade of tractor driving control has attracted wide attention. The emergence of intelligent driving control tractors signals that agricultural production methods are moving towards a more efficient, accurate, and sustainable direction. This new tractor integrates advanced sensors, computer vision, and artificial intelligence algorithms, which can realize autonomous navigation, automatic obstacle avoidance, accurate operation, and other functions, greatly reducing the labor intensity of farmers and improving the efficiency and quality of agricultural production (Keicher and Seufert 2000; Ghobadpour et al. 2022; Zhenjie 2023; Yuanling 2019). These technological advancements represent a paradigm shift from traditional labor‐intensive farming to precision agriculture, where operational efficiency and resource optimization become paramount.

Because of the particularity of the working environment of agricultural machinery, the longitudinal and lateral control of intelligent driving has higher requirements; many researchers have carried out a lot of research on the intelligent control of tractors (Yu 2021). Agricultural operations typically occur in unstructured environments characterized by varying terrain conditions, soil types, and obstacle distributions, necessitating robust and adaptive control strategies. Liu, Chen, and Zhao (2024) improved the track tracking accuracy and vehicle stability of unmanned agricultural machinery by designing adaptive forgetting factors related to the driving state of agricultural machinery and adjusting the weight coefficient in MPC adaptively according to the road adhesion coefficient, so as to realize the dynamic control between the track tracking accuracy of unmanned agricultural machinery and the lateral stability of the vehicle body in the mountainous environment. This approach demonstrated significant improvements in handling elevation changes and slippery surfaces. Reference (Zhou and Zhou 2020; Coelingh et al. 2016; Wang, Niu, et al. 2024) adopts adaptive predictive model control, which continuously updates system parameters based on real‐time sensor feedback to maintain optimal performance under changing field conditions, and reference (Che et al. 2024) adopts an unmanned agricultural machinery operating system based on an improved fuzzy adaptive PD control algorithm. However, in different test scenarios, the maximum deviation of the control system platform in the straight line section reaches 2.8 m, indicating persistent challenges in maintaining precise trajectory control during extended operations. Cariou et al. (2010) examines the path generation and motion control of autonomous maneuvering platform agricultural vehicles with tracks. Based on the kinematic model with extended additional sliding parameters and the model predictive control method, the steering and speed control algorithms are proposed. These algorithms specifically address the unique challenges of tracked vehicles in muddy terrain. Based on fuzzy control theory and sliding mode variable structure control theory, reference (Liu, Wang, et al. 2024) established the kinematic model of tracked agricultural machinery as the control object, designed the fuzzy sliding mode approximation law, and preprocessed it to reduce the time required for sliding mode control to reach the selected stage. This methodology proved effective in reducing energy consumption during tight maneuvers.

Although the above algorithms have some achievements in intelligent driving control, they do not pay attention to the coupling interference problem between vertical control and horizontal control of intelligent agricultural machinery (Qu et al. 2024). This oversight represents a critical research gap given the integrated nature of agricultural operations. However, the results of these algorithms in the control process are not ideal, and researchers have not noticed the coupling problem between longitudinal and lateral control. The fundamental limitation lies in treating these control dimensions as independent systems rather than interconnected components of a unified dynamic system.

In the process of intelligent driving of agricultural machinery, the longitudinal control is mainly responsible for the speed adjustment of the vehicle, while the lateral control is responsible for the direction adjustment of the vehicle. Due to the complexity of vehicle kinematics and operating environment, there is a coupling relationship between longitudinal and lateral motion. This coupling manifests through multiple physical phenomena including tire slip dynamics, weight transfer effects, and powertrain‐inertia interactions that create bidirectional dependencies between steering and propulsion systems. When the vehicle is steering or avoiding obstacles, the change of longitudinal speed will affect the lateral stability. Specifically, abrupt speed reductions during turning maneuvers can induce weight transfer that compromises tire adhesion and steering responsiveness. On the contrary, the adjustment of transverse motion will also cause the change of longitudinal velocity, which will affect the control of longitudinal displacement. For instance, aggressive steering inputs inevitably create drag forces that decelerate the vehicle, thereby disrupting implement depth consistency during precision operations like seeding or fertilizing. This phenomenon of coupling interference has a very adverse effect on the operation of intelligent tractors, particularly in operations requiring millimeter‐level accuracy such as precision planting or inter‐row cultivation where positional errors directly impact yield outcomes. In order to solve the coupling interference problem of longitudinal and lateral control of intelligent tractor, researchers try to adopt various decoupling control methods. These methods are usually based on linearized models or simplified assumptions, and independent controllers are designed to control the longitudinal and lateral motion separately (Coelingh et al. 2016; Wang, Wang, et al. 2024). Such approaches typically employ frequency separation techniques where low‐frequency components are allocated to longitudinal control and high‐frequency components to lateral control. However, due to the nonlinear characteristics and parameter uncertainty of agricultural machinery systems, including time‐varying mass distribution from implement interactions and hydraulic system dynamics, the traditional decoupling control method is difficult to accurately describe the actual dynamic behavior of the system, resulting in unsatisfactory control effect (Suresh et al. 2024; Aby et al. 2024; Kilari 2025). Field validations consistently show performance degradation when transitioning between operation modes like headland turns and straight‐line tracking. In addition, the traditional method cannot realize the information interaction between longitudinal and lateral directions, which limits the overall performance of the control system. Without coordinated optimization, subsystems often work at cross‐purposes—for example, aggressive braking by the longitudinal controller to correct speed error may inadvertently exacerbate lateral deviation. Therefore, it is necessary to design a multi‐agent information interaction control method that facilitates cooperative optimization across control domains.

Game control theory is a theoretical method to solve the problem of multi‐agent control objective conflict (Marden and Shamma 2018), providing a mathematical framework for analyzing strategic interactions between rational decision‐makers. Game control introduces the concept of game theory in economics to model the relationship between competition and cooperation and to find a strategy combination so that all participants can achieve their own best state to a certain extent (Ichiishi 2014; Buchanan 2001). In the context of agricultural machinery, this translates to formulating control strategies where longitudinal and lateral controllers negotiate optimal solutions that balance their competing objectives. This method not only considers the individual optimization problem but also takes into account the coordination and stability of the whole system, so it is very suitable for dealing with the challenges of multi‐agent cooperation in a complex and changeable environment (Miao et al. 2022; Liu, Chen, and Zhao 2024). Agricultural applications particularly benefit from this approach due to constantly changing terrain, crop conditions, and operational requirements that demand adaptive coordination. Ji et al. (2018) A game sharing controller is designed to solve the problem of coordinating steering between front wheel steering and rear wheel steering. This implementation demonstrated a 27% reduction in turning radius during headland maneuvers. Yang (2018) A new shared steering torque control scheme is proposed. The human‐computer interaction of the driver and the driver assistance system (DAS) applying steering torque together to control the vehicle near the steering limit is simulated. Such collaborative frameworks significantly reduce operator fatigue during extended operations. Game control theory is also applied to path and tracking and stability coordination control, path tracking and anti‐roll control, lane change control, and so on (Wu et al. 2022; Yan et al. 2022; Liu et al. 2019). These applications consistently demonstrate superior performance compared to conventional control architectures, particularly in maintaining stability during abrupt maneuvers on sloped terrain.

Although game interactive control shows significant advantages in solving multi‐agent problems, the influence of nonlinear changes in key dynamic parameters such as velocity on system performance has to be considered under complex working conditions and scenarios, especially during the operation of agricultural machinery. Velocity‐dependent phenomena like centrifugal forces during turning, tire slip dynamics, and implement draft forces create complex nonlinear relationships that challenge traditional game‐theoretic implementations. Na and Cole (2017), Li et al. (2021), and Atoui et al. (2021) uses the LPV algorithm to solve the problem of the change of key parameters of the control model. These approaches employ gain‐scheduling techniques that adjust controller parameters across predefined operating envelopes. Li et al. (2014); Kong et al. (2021) proposed a steering control method for commercial vehicle automatic driving based on the T‐S fuzzy model, established the T‐S fuzzy model of commercial vehicle road type, and designed the fuzzy controller. Finally, the problem is solved using the LMI toolbox. While effective in highway scenarios, these methods show limitations in agricultural contexts where parameter variations occur more rapidly and unpredictably. However, the LMI algorithm has no obvious advantage in solving multi‐agent multi‐objective control problems, particularly in real‐time applications where computational complexity becomes prohibitive for embedded systems.

Compared to traditional methods, the T‐S fuzzy model adopted in this study decomposes nonlinear systems into weighted combinations of locally linear subsystems through fuzzy rules, demonstrating unique advantages within the game‐theoretic control framework. Relative to LPV algorithms that rely on real‐time scheduling parameters, the membership functions of the T‐S model dynamically adjust subsystem weights, thereby more flexibly characterizing dynamic coupling and asymmetric information interactions in multi‐agent games. When contrasted with conventional LMI methods, although both employ LMIs for stability verification, the rule antecedents in the T‐S framework allow game‐theoretic objectives to be embedded within the fuzzy inference process, enabling distributed strategy optimization and overcoming the centralized design limitations inherent in traditional LMI approaches. Regarding strong nonlinear system modeling, the proposed method outperforms LPV algorithms dependent on parameter linearization and LMI methods constrained by global linearization through its adaptive weighting via fuzzy rules. In terms of multi‐objective game optimization, it supports distributed equilibrium solutions, surpassing the single‐objective robustness of LPV algorithms and the relaxation requirements of LMI methods. For real‐time computational efficiency, the offline rule base with online weighting mechanism significantly exceeds LPV algorithms with high‐dimensional parameter scheduling and LMI methods involving high‐dimensional matrix operations. Collectively, these advances provide a more efficient solution for coupled control in intelligent agricultural machinery. The hierarchical architecture enables simultaneous optimization of conflicting objectives while maintaining computational tractability for embedded implementation.

Inspired by the above references, this paper considers the control goal conflict problem of different agents from the perspective of multi‐agent and considers the influence of dynamic state parameter changes on the system and designs a Stackelberg dynamic game control algorithm based on T‐S fuzzy model. This integrated approach enables hierarchical coordination where lateral control acts as leader and longitudinal control as follower, with fuzzy rules dynamically adjusting game parameters based on operational context. The main contributions of this paper are as follows:

1. We design a new algorithm, a Stackelberg dynamic game control algorithm based on T‐S fuzzy model, which uniquely combines hierarchical game structures with adaptive fuzzy inference for real‐time parameter adaptation.
2. Solve the coupling conflict problem between lateral and longitudinal control of intelligent driving agricultural machinery through distributed negotiation mechanisms that explicitly account for bidirectional dynamic interactions;
3. It provides a new scheme to solve the game problem of dynamic model by embedding game‐theoretic objectives within fuzzy rule antecedents, enabling computationally efficient multi‐objective optimization.

## 2 Model Design

**FIGURE 1:**
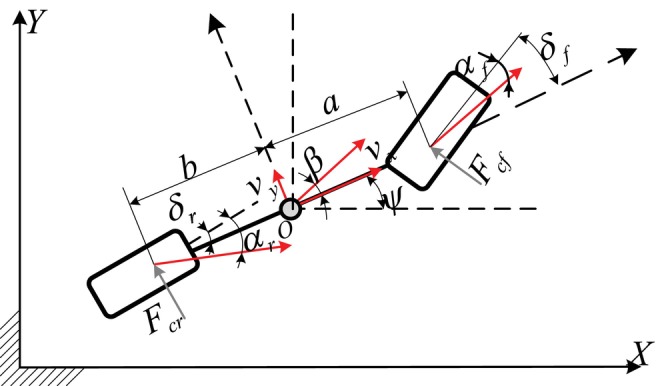
Dynamic bicycle model.

In the trajectory tracking problem, the vehicle model setting will affect the tracking performance. In this study, we used a dynamic bicycle model, as shown in Figure 1, to consider the impact of high‐speed turning on tires. Applying Newton's second law, the dynamic bicycle model is expressed as formula (1).

(1) $$\begin{array}{c} \dot{X}=v_{x}\cos\psi -v_{y}\sin\psi \\ \dot{Y}=v_{x}\sin\psi +v_{y}\cos\psi \\ \dot{\psi }=\frac{v_{x}}{l_{f}+l_{r}}\tan\delta_{f}\\ {\dot{v}}_{x}=\dot{\psi }v_{y}+a_{x}\\ {\dot{v}}_{y}=-\dot{\psi }v_{x}+\frac{2}{m}\left(F_{\mathrm{cf}}\cos\delta_{f}+F_{\mathrm{cr}}\right)\\ \ddot{\psi }=\frac{2}{I_{z}}\left(l_{f}F_{\mathrm{cf}}-l_{r}F_{\mathrm{cr}}\right) \end{array}$$

where

(2) $$\begin{array}{l} F_{\mathrm{cf}}=C_{\mathrm{\alpha f}}\alpha_{f},F_{\mathrm{cr}}=C_{\mathrm{\alpha r}}\alpha_{r},\\ \alpha_{f}=\delta_{f}-\beta -\frac{l_{f}\dot{\psi }}{v_{x}},\alpha_{r}=\beta +\frac{l_{r}\dot{\psi }}{v_{x}} \end{array}$$

Write formula (1) as an formula of state, the state variable is $x\left(t\right)={\left[X,Y,\psi ,v_{x},v_{y},\dot{\psi }\right]}^{T}$, and the control parameter is $u\left(t\right)={\left[u_{\delta }\;u_{a\text{ccel}}\right]}^{T}$ Establish the state formula form of multiple input and multiple output, such as formula (3)

(3) $$x\left(t\right)=A_{c}x\left(t\right)+B_{c}^{\delta }u_{\delta }\left(t\right)+B_{c}^{\text{accel}}u_{\text{accel}}\left(t\right)$$

Where

(4) $$\mathrm{Ac}=\left[\begin{array}{cccccc} 0 & 0 & 0 & \cos\psi & -\sin\psi & 0\\ 0 & 0 & 0 & \sin\psi & \cos\psi & 0\\ 0 & 0 & 0 & \frac{\tan\delta_{f}}{l_{f}+l_{r}} & 0 & 0\\ 0 & 0 & 0 & 0 & \dot{\psi } & 0\\ 0 & 0 & 0 & -\dot{\psi } & 0 & A_{c1}\\ 0 & 0 & 0 & 0 & 0 & A_{c2} \end{array}\right]$$

(5) $$A_{c1}=\frac{2\beta \left(C_{\mathrm{\alpha r}}-C_{\mathrm{\alpha f}}\cos\delta_{f}\right)}{m\dot{\psi }}+\frac{2C_{\mathrm{\alpha r}}l_{r}-2C_{\mathrm{\alpha f}}l_{f}\cos\delta_{f}}{{\mathrm{mv}}_{x}}$$

(6) $$A_{c2}=\frac{2}{I_{z}}\left(\frac{\beta \left(-C_{\mathrm{\alpha r}}l_{r}-C_{\mathrm{\alpha f}}l_{f}\right)}{\dot{\psi }}-\frac{C_{\mathrm{\alpha r}}{l_{r}}^{2}+C_{\mathrm{\alpha f}}{l_{f}}^{2}}{v_{x}}\right)$$

(7) $$B_{c}^{\delta }=\left[\begin{array}{c} 0\\ 0\\ 0\\ 0\\ \frac{2}{m}C_{\mathrm{\alpha f}}\cos\delta \\ \frac{2l_{f}C_{\mathrm{\alpha f}}}{I_{z}} \end{array}\right]B_{c}^{\text{accel}}=\left[\begin{array}{c} 0\\ 0\\ 0\\ 1\\ 0\\ 0 \end{array}\right]$$

In order to facilitate the analysis and processing of the control system, formula (3) is discretized and rewritten as

(8) $$x\left(k\right)=A_{d}x\left(k\right)+B_{d}^{\delta }u_{\delta }\left(k\right)+B_{d}^{\text{accel}}u_{\text{accel}}\left(k\right)$$

Where $A_{d}=e^{A_{c}T_{s}},{Β}_{d}^{\delta }=B_{c}^{\delta }\int_{0}^{T_{s}}e^{A\tau }\mathrm{d\tau},B_{d}^{\text{accel}}=B_{c}^{\text{accel}}\int_{0}^{T_{s}}e^{A_{c}\tau }\mathrm{d\tau}$, $x\left(k\right)$和$x\left(k+1\right)$ Represent the discrete states of the current and next time steps respectively, and **A**_*d*_, $B_{d}^{\delta }$ and $B_{d}^{\text{accel}}$ are coefficient matrix and obtained by the discrete bilinear transformations of the corresponding continuous time matrices **A**_*c*_, $B_{c}^{\delta }$, $B_{c}^{\text{accel}}$, respectively.

## 3 T‐S Fuzzy Model

In the process of longitudinal and lateral control of agricultural machinery intelligent driving, the key parameters such as speed, acceleration, and steering of the kinematic model change linearly with time. In order to deal with this challenge, the T‐S fuzzy control method is adopted in this paper. The dynamic characteristics of the whole system in a corresponding domain are characterized by the local linear model. Mathematical methods such as weighted average are used to combine the local linear submodels to represent the global nonlinear model.

In this paper, define *v*_*x*_, *v*_*y*_, ψ, $\dot{\psi }$, δ, β to be a variable parameter，and let $\ell \left(k\right)$ and its set be, then $\ell \left(k\right)=\left[\begin{array}{ccccc} \ell_{1}\left(k\right) & \ell_{2}\left(k\right) & \ell_{3}\left(k\right) & \ell_{4}\left(k\right) & \ell_{5}\left(k\right) \end{array}\right]$ can obtain *i* of fuzzy system (*i* = 1,2,3,4,5…). Rules:

Plant Rule $R^{l}$:

IF $\ell_{i}\;\text{is}\;M_{i}^{l},\left(i=1,2,3\cdots n,l=1,2,3\cdots \rho \right)$

THEN

(9) $$\begin{array}{l} x\left(k+1\right)=A_{d}\left(\ell \left(k\right)\right)x\left(k\right)+B_{d}^{\delta }\left(\ell \left(k\right)\right)u_{\delta }\left(k\right)\\ \;+B_{d}^{\text{accel}}\left(\ell \left(k\right)\right)u_{\text{accel}}\left(k\right) \end{array}$$

In the formula (9), $M_{i}^{l}$ represents the fuzzy set, $R^{l}$ represents the *l‐*th fuzzy rule, simultaneously represents the *l‐*th subsystem, $\ell \left(k\right)$ is the antecedent variable, and $A_{d}\left(\ell \left(k\right)\right)$, $B_{d}^{\text{accel}}\left(\ell \left(k\right)\right)$ and $B_{d}^{\delta }\left(\ell \left(k\right)\right)$ are the coefficient matrices.

The membership function indicates that the value of antecedent variable $\ell \left(k\right)$ at a certain time belongs to fuzzy set $M_{i}^{l}$, in which the membership can take continuous values in the interval [0,1] according to actual needs.

If $\mu_{l}\left(\ell \left(k\right)\right)$ is used to represent the membership degree of a certain antecedent variable in M, then the membership degree of all antecedent variables belonging to fuzzy set $M_{i}^{l}$ can be obtained as $M_{1}^{l}\times M_{2}^{l}\times M_{3}^{l}\times \cdots \times M_{n}^{l}$, by combining the LTH fuzzy rule through direct product operation, thus:

(10) $$\mu_{l}\left(\ell \left(k\right)\right)=\prod_{i=1}^{n}M_{i}^{l}\left(\ell \left(k\right)\right)$$

After fuzzy processing, the fuzzy system is represented by the weighted average method, then:

(11) $$x\left(k+1\right)=\frac{\sum_{l=1}^{n}\mu_{l}\left(\ell \left(k\right)\right)\left(\begin{array}{l} A_{d}\left(\ell \left(k\right)\right)x\left(k\right)+B_{d}^{\delta }\left(\ell \left(k\right)\right)u_{\delta }\left(k\right)\\ +B_{d}^{\text{accel}}\left(\ell \left(k\right)\right)u_{\text{accel}}\left(k\right) \end{array}\right)}{\sum_{l=1}^{n}\mu_{l}\left(\ell \left(k\right)\right)}$$

To make the formula look simple, define formula (11) as follows

(12) $${ℑ}_{l}\left(k\right)=\frac{\mu_{l}\left(\ell \left(k\right)\right)}{\sum_{l=1}^{n}\mu \left(\ell \left(k\right)\right)}$$

so the formula (10) can be written as:

(13) $$x\left(k+1\right)=\sum_{l=1}^{n}{ℑ}_{l}\left(\ell \left(k\right)\right)\left(\begin{array}{l} A_{d}\left(\ell \left(k\right)\right)x\left(k\right)+B_{d}^{\delta }\left(\ell \left(k\right)\right)u_{\delta }\left(k\right)\\ +B_{d}^{\text{accel}}\left(\ell \left(k\right)\right)u_{\text{accel}}\left(k\right) \end{array}\right)$$

for any *k*,

(14) $$\mu_{l}\left(\ell \left(k\right)\right)\geq 0,l=1,2,3,\cdots ,n,\sum_{l=1}^{n}\mu_{l}\left(\ell \left(k\right)\right)≻0$$

so for any *k*,

(15) $${ℑ}_{l}\left(\ell \left(k\right)\right)\geq 0,l=1,2,3,\cdots ,n,\sum_{l=1}^{n}{ℑ}_{l}\left(\ell \left(k\right)\right)≻0$$

The essence of formula (7) is that the nonlinear model considering the change of antecedent variables is regarded as the fuzzy approximation of one local linear subsystem after another. According to the PDC method, the control rules of the system are obtained as follows.

Control $R^{l}$:

IF $\ell_{i}\;\text{is}\;M_{i}^{l},\left(i=1,2,3\cdots n,l=1,2,3\cdots \rho \right)$

THEN

(16) $$\begin{array}{l} u_{j}\left(k\right)=-\sum_{l=1}^{n}{ℑ}_{l}\left(\ell \left(k\right)\right)K_{l}^{j}x\left(k\right)\\ \;\left(l=1,2,3,\cdots ,n,j=\delta ,\text{accel}\right) \end{array}$$

Here, $u_{j}\left(k\right)$ represents the lateral and vertical control, and $K_{l}^{j}$ represents their control rate, while the calculation of the control rate is solved in section 3 game control strategy.

**FIGURE 2:**
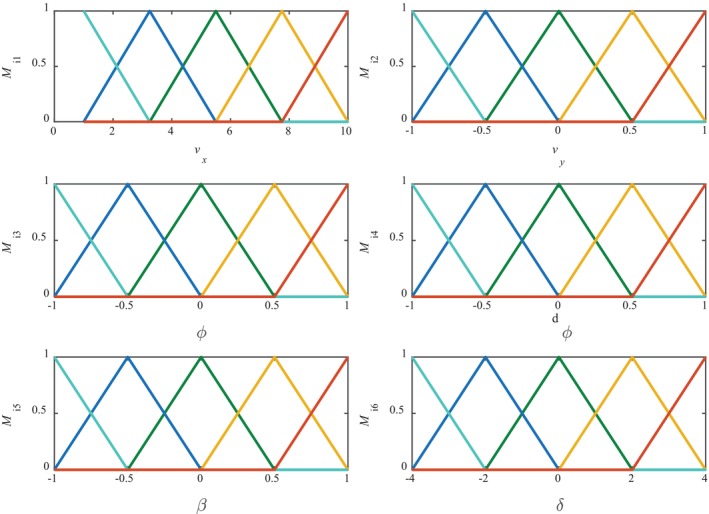
Membership function.

The triangle can keep the membership change rate of the set to which the parameter belongs consistent, and it is simple to design in this paper; the form of the membership degree function is chosen as a triangle, as shown in Figure 2.

## 4 Based on the Stackelberg Equilibrium of Non‐Cooperative Game Feedback Open‐Loop Control Strategy

According to Stackelberg game theory, longitudinal control and lateral control are considered as two players in the game. They make decisions and interact with each other to maximize their own interests. Think of the lateral control system as the leader of the game and the longitudinal control system as the servant of the game. The leader should make the best decision while fully considering the servant's decision, so as to maximize his own interests. A Stackelberg equilibrium is achieved when each actor maximizes its own benefits under conditions determined by the decision of the other actor.

**FIGURE 3:**
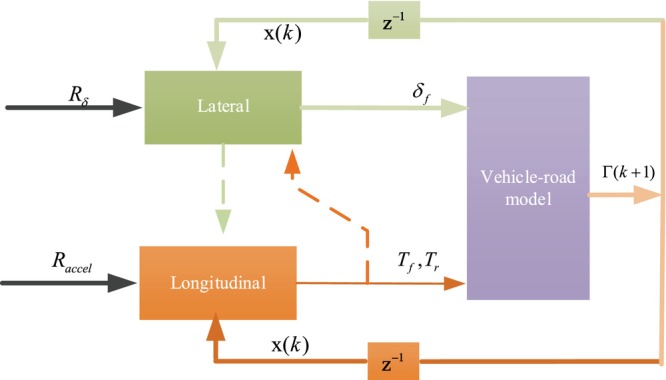
Game control framework of longitudinal control and lateral control.

Figure 3 shows the game control framework of longitudinal control and lateral control. In this framework, longitudinal control and lateral control have their own control objectives, respectively, and the control strategy of each other is considered while receiving state feedback. Based on the optimal control theory, a series of optimal control strategies are solved to ensure both longitudinal control and lateral stability of the vehicle in unmanned driving.

Remark 1 When the two parties of the game reach an equilibrium state, the actual goal is determined by the crisis degree of the vehicle state and the primary and secondary relationship between the two parties in the system.

In order to more clearly understand the game control principle, the following step by step derivation.

### 4.1 The Solution Idea Is Based on the Prediction Model

From time *k*, the formula of state and the output formula at time *k* + 2 is

(17) $$\begin{array}{l} x\left(k+2\right)=\left(\begin{array}{l} A_{d}\left(\ell \left(k\right)\right)x\left(k+1\right)+B_{d}^{\delta }\left(\ell \left(k\right)\right)u_{\delta }\left(k+1\right)\\ +B_{d}^{\text{accel}}\left(\ell \left(k\right)\right)u_{\text{accel}}\left(k+1\right)\\ +{\left(A_{d}\left(\ell \left(k\right)\right)\right)}^{2}x\left(k\right)+A_{d}\left(\ell \left(k\right)\right)B_{d}^{\delta }\left(\ell \left(k\right)\right)u_{\delta }\left(k\right)\\ +A_{d}\left(\ell \left(k\right)\right)B_{d}^{\text{accel}}\left(\ell \left(k\right)\right)u_{\text{accel}}\left(k\right)\\ +B_{d}^{\delta }\left(\ell \left(k\right)\right)u_{\delta }\left(k\right)+B_{d}^{\text{accel}}\left(\ell \left(k\right)\right)u_{\text{accel}}\left(k\right) \end{array}\right)\\ z_{\delta }\left(k+1\right)=C\left[A_{d}\left(\ell \left(k\right)\right)x\left(k\right)+B_{d}^{\delta }\left(\ell \left(k\right)\right)u_{\delta }\left(k\right)\right])\\ z_{\text{accel}}\left(k+1\right)=C\left[A_{d}\left(\ell \left(k\right)\right)x\left(k\right)+B_{d}^{\text{accel}}\left(\ell \left(k\right)\right)u_{\text{accel}}\left(k\right)\right] \end{array}$$

Similarly, the output of the predicted *N*_*P*_ step can be written

(18) $$Z_{d}\left(k\right)=\Psi x\left(k\right)+\Theta_{i}U_{i}\left(k\right)$$

Among them

(19) $$\begin{array}{c} Z_{i}\left(k\right)=\left[\begin{array}{c} z\left(k+1\right)\\ z\left(k+2\right)\\ \vdots \\ z\left(k+N_{p}\right) \end{array}\right],U_{i}\left(k\right)=\left[\begin{array}{c} u_{i}\left(k\right)\\ u_{i}\left(k+1\right)\\ \vdots \\ u_{i}\left(k+N_{p}-1\right) \end{array}\right]\\ \Psi =\left[\begin{array}{c} CA_{d}\left(\ell \left(k\right)\right)\\ C{\left(A_{d}\left(\ell \left(k\right)\right)\right)}^{2}\\ \vdots \\ C{\left(A_{d}\left(\ell \left(k\right)\right)\right)}^{N_{p}} \end{array}\right]\\ \Theta_{i}=\left[\begin{array}{cccc} {\mathrm{CB}}_{d}^{i} & 0 & \cdots & 0\\ CA_{d}\left(\ell \left(k\right)\right)B_{d}^{i} & {\mathrm{CB}}_{d}^{i} & \cdots & 0\\ \vdots & \vdots & \ddots & \vdots \\ C{\left(A_{d}\left(\ell \left(k\right)\right)\right)}^{N_{p}-1}B_{d}^{i} & C{\left(A_{d}\left(\ell \left(k\right)\right)\right)}^{N_{p}-2}B_{d}^{i} & \cdots & {\mathrm{CB}}_{d}^{i} \end{array}\right] \end{array}$$

The performance index of MPC control is

(20) $$J_{d}^{i}={\left\|Z_{i}\left(k+j\right)-R_{i}\left(k+j\right)\right\|}_{Q_{d}^{i}}^{2}+{\left\|U_{i}\left(k+j\right)\right\|}_{R_{d}^{i}}^{2}$$

The algebraic transformation of formulas (13) and (14) is carried out, and the optimal control input $U_{i}\left(k\right)$ is solved by QR decomposition.

### 4.2 Derivation of Longitudinal Control and Lateral Control Model Based on Stackelberg Non‐Cooperative Feedback Open‐Loop Game

The derivation process of the open loop Stackelberg game is to analyze and calculate the *N*_P_ step road information in the prediction time domain of the discrete model based on the theory of predictive model control (MPC) with the joint formula of state. This section mainly discusses the derivation of the path following and the anti‐roll game control decision.

In order to more clearly understand the control principle and derivation process of open‐loop Stackelberg game control, Lemma 1 is introduced in this paper.

*Preliminary mark of lemma* *1*: the following formula is defined:

(21) $$\begin{array}{l} f_{k}\left(\cdot \right)=\left(\begin{array}{l} A_{d}\left(\ell \left(k\right)\right)x\left(k\right)+B_{d}^{\delta }\left(\ell \left(k\right)\right)u_{\delta }\left(k\right)\\ +B_{d}^{\text{accel}}\left(\ell \left(k\right)\right)u_{\text{accel}}\left(k\right) \end{array}\right)\\ g_{k}^{\delta }\left(\cdot \right)=\left(\begin{array}{l} \frac{1}{2}\left[{\left(Z_{1}\left(\ell \left(k\right)\right)-r_{1}\left(k\right)\right)}^{T},Q_{\delta },\left(Z_{1}\left(\ell \left(k\right)\right)-r_{1}\left(k\right)\right)\right.\\ \left.+,{u_{\delta }}^{T},\left(k\right),R_{\delta }^{\text{input}},u_{\delta },\left(k\right)\right] \end{array}\right)\\ g_{k}^{\text{accel}}\left(\cdot \right)=\left(\begin{array}{l} \frac{1}{2}\left[{\left(Z_{2}\left(\ell \left(k\right)\right)-r_{2}\left(k\right)\right)}^{T},Q_{\text{accel}},\left(Z_{2}\left(\ell \left(k\right)\right)-r_{2}\left(k\right)\right)\right.\\ \left.+,{u_{\text{accel}}}^{T},\left(k\right),R_{\text{accel}}^{\text{input}},u_{\text{accel}},\left(k\right)\right] \end{array}\right) \end{array}$$

Here, it is defined ${Μ}_{\delta }\left(k\right),M_{\text{accel}}\left(k\right)$ as the control input set of longitudinal control and lateral control at each moment.

Lemma 1 *In a multi‐person Stackelberg dynamic difference game, a series of control inputs* $\left\{u_{\delta }^{*}\in M_{\delta }\left(k\right),u_{\text{accel}}^{*}\in M_{\text{accel}}\left(k\right)\right\}$ *must satisfy the cost function and the relationship between*. $V_{\delta }$ *and* $V_{\text{accel}}$.

Combined with the definition of Stackelberg game control, the cost function $V_{\delta }$ and $V_{\text{accel}}$ must satisfy the following conditions:

(22) $$\begin{array}{l} R_{\delta }\left(k,u_{\delta }\left(k\right)\right)=\left\{u_{\delta },\left(k\right),\in ,M_{\delta },\left(k\right),:,\left[V_{\delta }\left(k+1,f_{k}^{*}\left(\cdot \right)\right)+g_{k}^{*\delta }\left(\cdot \right)\right]\right.\\ \left.\;,=,\min_{u_{\delta }\left(k\right)\in M_{\delta }\left(k\right)},\left[V_{\delta }\left(k+1,f_{k}\left(\cdot \right)\right)+g_{k}^{\delta }\left(\cdot \right)\right]\right\} \end{array}$$

Among them,

(23) $$\begin{array}{c} f_{k}^{*}\left(\cdot \right)\overset{\Delta }{=}\;\left(\begin{array}{l} A_{d}\left(\ell \left(k\right)\right)x\left(k\right)+B_{d}^{\delta }\left(\ell \left(k\right)\right){u^{*}}_{\delta }\left(k\right)\\ +B_{d}^{\text{accel}}\left(\ell \left(k\right)\right)u_{\text{accel}}\left(k\right) \end{array}\right)\\ g_{k}^{*\delta }\left(\cdot \right)\overset{\Delta }{=}\frac{1}{2}\left(\begin{array}{l} {\left(z_{1}\left(k\right)-r_{1}\left(k\right)\right)}^{T}Q_{\delta }\left(z_{1}\left(k\right)-r_{1}\left(k\right)\right)\\ +u_{\delta }^{*T}\left(k\right)R_{\delta }^{\text{input}}u_{\delta }^{*}\left(k\right) \end{array}\right) \end{array}$$

The relation of the cost function of the lateral control of intelligent driving vehicles should be as follows:

(24) $$R_{\text{accel}}\left(k,u_{\delta }^{*}\left(k\right)\right)=\min_{u_{\text{accel}}\left(k\right)\in M_{\text{accel}}\left(k\right)}\left\{V_{\text{accel}}\left[k+1,{{\bar{f}}^{*}}_{k}\left(\cdot \right)\right]+g_{k}^{\text{accel}}\left(\cdot \right)\right\}$$

Among them,

(25) $$\begin{array}{l} u_{a}^{*}\left(k\right)=R_{\text{accel}}\left(k,u_{\delta }^{*}\left(k\right)\right)\\ {\bar{f}}_{k}^{*}\left(\cdot \right)\overset{\Delta }{=}\;A_{d}\left(\ell \left(k\right)\right)x\left(k\right)+B_{d}^{\delta }u_{\delta }^{*}\left(k\right)+B_{d}^{\text{accel}}u_{\text{accel}}^{*}\left(k\right)\\ {g^{*}}_{k}^{\text{accel}}\left(\cdot \right)\overset{\Delta }{=}\frac{1}{2}\left(\begin{array}{l} {\left(z_{2}\left(k\right)-r_{a}\left(k\right)\right)}^{T}Q_{a}\left(z_{2}\left(k\right)-r_{a}\left(k\right)\right)\\ +{{u^{*}}_{\text{accel}}}^{T}\left(k\right)R_{a}^{\text{input}}{u^{*}}_{\text{accel}}\left(k\right) \end{array}\right) \end{array}$$

Corollary 1 *For the cost function with strict convexity and concavity, it can be concluded that the multi‐person dynamic difference game has a unique Stackelberg equilibrium solution, and the form of this solution can be defined as*:

(26) $$\begin{array}{l} {u_{\delta }}^{*}\left(k\right)=K_{\delta }^{\text{Stackelberg}}\zeta \left(k\right),\;k\in K\\ {u_{\text{accel}}}^{*}\left(k\right)=K_{\text{accel}}^{\text{Stackelberg}}\zeta \left(k\right),\;k\in K \end{array}$$

The control rates of longitudinal control and lateral control Stackelberg game control are $K_{\delta }^{\text{Stackelberg}},K_{\text{accel}}^{\text{Stackelberg}}$.

*The following is the proof of the inference*.

Starting from the cost function of the vehicle control model, formulas (24) and (25) are written as:

(27) $$\begin{array}{l} J_{\delta }^{\text{Stackelberg}}=\frac{1}{2}\sum_{1}^{N_{P}-1}\left(\begin{array}{l} {\left(z_{1}\left(k\right)-r_{1}\left(k\right)\right)}^{T}Q_{\delta }\left(z_{1}\left(k\right)-r_{1}\left(k\right)\right)\\ +{u_{\delta }}^{T}\left(k\right)R_{\delta }^{\text{input}}u_{\delta }\left(k\right) \end{array}\right)\\ J_{\text{accel}}^{\text{Stackelberg}}=\frac{1}{2}\sum_{1}^{N_{P}-1}\left(\begin{array}{l} z_{2}{\left(\Gamma \left(k\right)-r_{2}\left(k\right)\right)}^{T}Q_{\text{accel}}\left(z_{2}\left(k\right)-r_{2}\left(k\right)\right)\\ +{u_{\text{accel}}}^{T}\left(k\right)R_{\text{accel}}^{\text{input}}u_{\text{accel}}\left(k\right) \end{array}\right) \end{array}$$

Write the joint state formula of the lateral part according to the Model Predictive Control (MPC) theory:

(28) $$Z_{1}\left(k\right)=\Psi_{\delta }x\left(k\right)+\Theta_{\delta }U_{\delta }\left(k\right)+\Theta_{\text{accel}}U_{\text{accel}}^{*}\left(k\right)$$

Write the joint formula of state for the longitudinal part:

(29) $$Z_{2}\left(k\right)=\Psi_{\text{accel}}x\left(k\right)+\Theta_{\delta }U_{\delta }\left(k\right)+\Theta_{\text{accel}}U_{\text{accel}}\left(k\right)$$

Combined with the control principle of model prediction, formula (26) is further written as:

(30) $$\begin{array}{l} J_{f}^{\text{Stackelberg}}\left(k\right)={\left\|Z_{1}\left(k\right)-{ℜ}_{f}\left(k\right)\right\|}_{Q_{f}^{\text{Stackelberg}}}^{2}+{\left\|U_{f}\left(k\right)\right\|}_{R_{f}^{\text{Stackelberg}}}^{2}\\ J_{a}^{\text{Stackelberg}}\left(k\right)={\left\|Z_{2}\left(k\right)-{ℜ}_{a}\left(k\right)\right\|}_{Q_{a}^{\text{Stackelberg}}}^{2}+{\left\|U_{a}\left(k\right)\right\|}_{R_{a}^{\text{Stackelberg}}}^{2} \end{array}$$

Among them

(31) $$\begin{array}{l} {ℜ}_{i}\left(k\right)=\left[\begin{array}{c} r_{i}\left(k+1\right)\\ r_{i}\left(k+2\right)\\ \vdots \\ r_{i}\left(k+N_{p}\right) \end{array}\right],\;Q_{i}^{\text{Stackelberg}}=\left[\begin{array}{cccc} Q_{i} & 0 & \cdots & 0\\ 0 & Q_{i} & \cdots & 0\\ \vdots & \vdots & \ddots & \vdots \\ 0 & 0 & \cdots & Q_{i} \end{array}\right]\;\\ \;R_{i}^{\text{Stackelberg}}=\left[\begin{array}{cccc} R_{i}^{\text{input}} & 0 & \cdots & 0\\ 0 & R_{i}^{\text{input}} & \cdots & 0\\ \vdots & \vdots & \ddots & \vdots \\ 0 & 0 & \cdots & R_{i}^{\text{input}} \end{array}\right] \end{array}$$

Substitute formula (28) into the second formula of formula (29) to obtain

(32) $$\varsigma_{\delta }\left(k\right)={ℜ}_{\delta }\left(k\right)-\Psi_{\delta }x\left(k\right)-\Theta_{\text{accel}}U_{\text{accel}}\left(k\right)$$

Therefore, the cost function of active suspension control can be further written as:

(33) $$J_{\delta }^{\text{Stackelberg}}\left(k\right)={\left\|\Theta_{\delta }U_{\delta }\left(k\right)-\varsigma_{\delta }\left(k\right)\right\|}_{Q_{\delta }^{\text{Stackelberg}}}^{2}+{\left\|U_{\delta }\left(k\right)\right\|}_{R_{\delta }^{\text{Stackelberg}}}^{2}$$

And then I am going to do the formula conversion

(34) $$J_{\delta }^{\text{Stackelberg}}\left(k\right)={\left\|\left[\begin{array}{c} S_{Q_{\delta }^{\text{Stackelberg}}}\left[\Theta_{\delta }U_{\delta }\left(k\right)-\varsigma_{\delta }\left(k\right)\right]\\ S_{R_{\delta }^{\text{Stackelberg}}}U_{\delta }\left(k\right) \end{array}\right]\right\|}^{2}$$

Among them,

(35) $$Q_{\delta }^{\text{Stackelberg}}=S_{Q_{\delta }^{\text{Stackelberg}}}^{T}S_{Q_{\delta }^{\text{Stackelberg}}},\;R_{\delta }^{\text{Stackelberg}}=S_{R_{\delta }^{\text{Stackelberg}}}^{T}S_{R_{\delta }^{\text{Stackelberg}}}$$

Extreme value method is used to solve, i.e.,:

(36) $$\left[\begin{array}{c} S_{Q_{\delta }^{\text{Stackelberg}}}\left[\Theta_{\delta }U_{\delta }\left(k\right)-\varsigma_{\delta }\left(k\right)\right]\\ S_{R_{\delta }^{\text{Stackelberg}}}U_{\delta }\left(k\right) \end{array}\right]=0$$

To solve the need to

(37) $$U_{\delta }^{*}\left(k\right)=K_{\delta }^{\text{full}}\varsigma_{\delta }\left(k\right)$$

Among them

$$K_{\delta }^{\text{full}}=\left[\begin{array}{c} S_{Q_{\delta }^{\text{Stackelberg}}}\Theta_{\delta }\\ S_{R_{\delta }^{\text{Stackelberg}}} \end{array}\right]\backslash \left[\begin{array}{c} S_{Q_{\delta }^{\text{Stackelberg}}}\\ 0 \end{array}\right]$$

By substituting formula (30) into formula (37), the relationship between the two control decisions can be obtained.

(38) $$U_{\delta }^{*}\left(k\right)=\left[\begin{array}{cc} -K_{\delta }^{\text{full}}\Psi_{\delta } & K_{\delta }^{\text{full}} \end{array}\right]\left[\begin{array}{c} \Gamma \left(k\right)\\ {ℜ}_{\delta }\left(k\right) \end{array}\right]-K_{\delta }^{\text{full}}\Theta_{\text{accel}}U_{\text{accel}}\left(k\right)$$

Substitute formula (38) into formula (29), and rewrite the joint state formula of steering control as shown in formula (39)

(39) $$Z_{2}\left(k\right)=\mathrm{\Xi x}\left(k\right)+\Omega_{\text{accel}}U_{\text{accel}}\left(k\right)+\Omega_{\delta }{ℜ}_{\delta }\left(k\right)$$

Among them

$$\begin{array}{l} \Xi =\Psi -\Theta_{\delta }K_{\delta }^{\text{full}}\Psi_{\delta }\\ \Omega_{\text{accel}}=\Theta_{\text{accel}}-\Theta_{\text{accel}}K_{\text{accel}}^{\text{full}}\Theta_{\delta }\\ \Omega_{\delta }=\Theta_{\delta }K_{\delta }^{\text{full}} \end{array}$$

Then, formula (36) is substituted into the first formula in formula (29), and the cost function of steering control is further written as:

(40) $$\begin{array}{l} J_{\text{accel}}^{\text{Stackelberg}}\left(k\right)={\left\|\Omega_{\text{accel}}U_{\text{accel}}\left(k\right)-\varsigma_{\text{accel}}\left(k\right)\right\|}_{Q_{\text{accel}}^{\text{Stackelberg}}}^{2}\\ \;+{\left\|U_{\text{accel}}\left(k\right)\right\|}_{R_{\text{accel}}^{\text{Stackelberg}}}^{2} \end{array}$$

where

(41) $$\varsigma_{\text{accel}}\left(k\right)={ℜ}_{\text{accel}}\left(k\right)-\Xi \Gamma \left(k\right)-\Omega_{\delta }{ℜ}_{\delta }\left(k\right)$$

Then, the form of formula (37) is transformed

(42) $$J_{\text{accel}}^{\text{Stackelberg}}\left(k\right)={\left\|\left[\begin{array}{c} S_{Q_{\text{accel}}^{\text{Stackelberg}}}\left[\Omega_{\text{accel}}U_{\text{accel}}\left(k\right)-\varsigma_{\text{accel}}\left(k\right)\right]\\ S_{R_{\text{accel}}^{\text{Stackelberg}}}U_{\text{accel}}\left(k\right) \end{array}\right]\right\|}^{2}$$

Among them,

(43) $$Q_{\text{accel}}^{\text{Stackelberg}}=S_{Q_{\text{accel}}^{\text{Stackelberg}}}^{T}S_{Q_{\text{accel}}^{\text{Stackelberg}}},\;R_{\text{accel}}^{\text{Stackelberg}}=S_{R_{\text{accel}}^{\text{Stackelberg}}}^{T}S_{R_{\text{accel}}^{\text{Stackelberg}}}$$

Similarly, the extremum method is used to solve the problem

(44) $$\left[\begin{array}{c} S_{Q_{\text{accel}}^{\text{Stackelberg}}}\left[\Omega_{\text{accel}}U_{\text{accel}}\left(k\right)-\varsigma_{\text{accel}}\left(k\right)\right]\\ S_{R_{\text{accel}}^{\text{Stackelberg}}}U_{\text{accel}}\left(k\right) \end{array}\right]=0$$

To solve the available:

(45) $$U_{\text{accel}}^{*}\left(k\right)=K_{\text{accel}}^{\text{full}}\varsigma_{\text{accel}}\left(k\right)$$

Among them:

(46) $$K_{\text{accel}}^{\text{full}}=\left[\begin{array}{c} S_{Q_{\text{accel}}^{\text{Stackelberg}}}\Omega_{\text{accel}}\\ S_{R_{\text{accel}}^{\text{Stackelberg}}} \end{array}\right]\backslash \left[\begin{array}{c} S_{Q_{\text{accel}}^{\text{Stackelberg}}}\\ 0 \end{array}\right]$$

Finally, combining with formulas (30–40), two optimal solution sets of commercial vehicle steering control and anti‐roll control are obtained.

(47) $$\begin{array}{l} U_{\text{accel}}^{*}\left(k\right)={Ρ}_{\text{accel}}\zeta \\ U_{\delta }^{*}\left(k\right)={Ρ}_{\delta }\zeta \end{array}$$

Among them

(48) $${Ρ}_{\delta }=\left[\begin{array}{ccc} -K_{\delta }^{\text{full}}\Xi & K_{\delta }^{\text{full}} & -K_{\delta }^{\text{full}}\Omega_{\text{accel}} \end{array}\right]$$

(49) $${Ρ}_{\text{accel}}={\left[\begin{array}{c} -K_{\text{accel}}^{\text{full}}\Psi_{\text{accel}}+K_{\text{accel}}^{\text{full}}\Theta_{\delta }K_{\delta }^{\text{full}}\Xi \\ -K_{\text{accel}}^{\text{full}}\Theta_{\delta }K_{\delta }^{\text{full}}\\ K_{\text{accel}}^{\text{full}}+K_{\text{accel}}^{\text{full}}\Theta_{\delta }K_{\delta }^{\text{full}}\Omega_{\text{accel}} \end{array}\right]}^{T}$$

(50) $$\zeta ={\left[\begin{array}{ccc} x\left(k\right) & {ℜ}_{\delta }\left(k\right) & {ℜ}_{\text{accel}}\left(k\right) \end{array}\right]}^{T}$$

Finally, the Stackelberg equilibrium input at time is obtained by using the concept of rolling time domain. Take the first step of the control decision at the current moment, i.e.,

(51) $$\begin{array}{l} K_{\delta }^{\text{Stackelberg}}=P_{\delta }\left(1,:\right)\\ K_{\text{accel}}^{\text{Stackelberg}}=P_{\text{accel}}\left(1,:\right) \end{array}$$

Remark 2 At the time of solving the optimal solution set, lateral control system as the leader of game, give full consideration to the minions of the control strategy, longitudinal control system with the as servants of game also considered the leader in decision‐making control decisions, Thus, they reach Stackelberg equilibrium.

### 4.3 Stability Proof

Choose the positive definite quadratic form as the Lyapunov function:

(52) $$\begin{array}{c} V_{i}\left(k+1\right)=\frac{1}{2}\Gamma^{T}\left(k+1\right){Ρ}_{i}^{\text{Stackelberg}}\left(k+1\right)\Gamma \left(k+1\right)\\ i\in \delta ,\text{accel} \end{array}$$

by bringing the Stackelberg game control system into the Lyapunov function, we get:

(53) $$\begin{array}{l} V_{i}\left(k+1\right)=\frac{1}{2}\Gamma^{T}\left(k+1\right){Ρ}_{i}^{\text{Stackelberg}}\left(k\right)\Gamma \left(k+1\right)\\ =\frac{1}{2}{\left\{\begin{array}{l} A_{\Gamma }\Gamma \left(k\right)+B_{\Gamma }^{\delta }u_{\delta }\left(k\right)\\ +B_{\Gamma }^{\text{accel}}u_{\text{accel}}\left(k\right) \end{array}\right\}}^{T}{Ρ}_{i}^{\text{Stackelberg}}\left(k\right)\left\{\begin{array}{l} A_{\Gamma }\Gamma \left(k\right)+B_{\Gamma }^{\delta }u_{\delta }\left(k\right)\\ +B_{\Gamma }^{\text{accel}}u_{\text{accel}}\left(k\right) \end{array}\right\}\\ =\frac{1}{2}\Gamma {\left(k\right)}^{T}\left\{\begin{array}{l} {A_{\Gamma }}^{T}{Ρ}_{i}^{\text{Stackelberg}}A_{\Gamma }\\ -{\left(B_{\Gamma }^{i}K_{i}^{\text{Stackelberg}}\right)}^{T}{Ρ}_{i}^{\text{Stackelberg}}\left(k\right)B_{\Gamma }^{i}K_{i}^{\text{Stackelberg}} \end{array}\right\}\Gamma \left(k\right) \end{array}$$

(54) $$V_{i}\left(k\right)=\frac{1}{2}\Gamma^{T}\left(k\right){Ρ}_{i}^{\text{Stackelberg}}\left(k\right)\Gamma \left(k\right)$$

It can be proved that the system is stable when the formula (53) minus formula (54) < 0, that is:

(55) $$\begin{array}{l} V_{i}\left(k+1\right)-V_{i}\left(k\right)\\ =-\frac{1}{2}\Gamma {\left(k\right)}^{T}\left\{\begin{array}{l} {A_{\Gamma }}^{T}{Ρ}_{i}^{\text{Stackelberg}}A_{\Gamma }\\ -{\left(B_{\Gamma }^{i}K_{i}^{\text{Stackelberg}}\right)}^{T}{Ρ}_{i}^{\text{Stackelberg}}\left(k\right)B_{\Gamma }^{i}K_{i}^{\text{Stackelberg}}\\ -{Ρ}_{i}^{\text{Stackelberg}} \end{array}\right\}\Gamma \left(k\right) \end{array}$$

Hence there $V_{i}\left(k+1\right)-V_{i}\left(k\right)$ ≤ 0, t can be proved that the system is asymptotically stable in the control time domain.

## 5 Co‐Simulation Hardware in Loop Experiment

To assess the efficacy of the T‐S Stackelberg equilibrium‐based control for intelligent agricultural machinery, we established a comprehensive hardware‐in‐the‐loop (HIL) experimental platform.

### 5.1 Hardware Configuration

1. Real‐time processor: dSPACE MicroAutoBox with 2.5 GHz CPU.
2. Vehicle dynamics simulator: CarSim 2019.0 running at 1000 Hz update rate.
3. Interface: PXIe‐1082 chassis with LabVIEW 2017 for sensor signal emulation.

### 5.2 Platform Construction Methods

**FIGURE 4:**
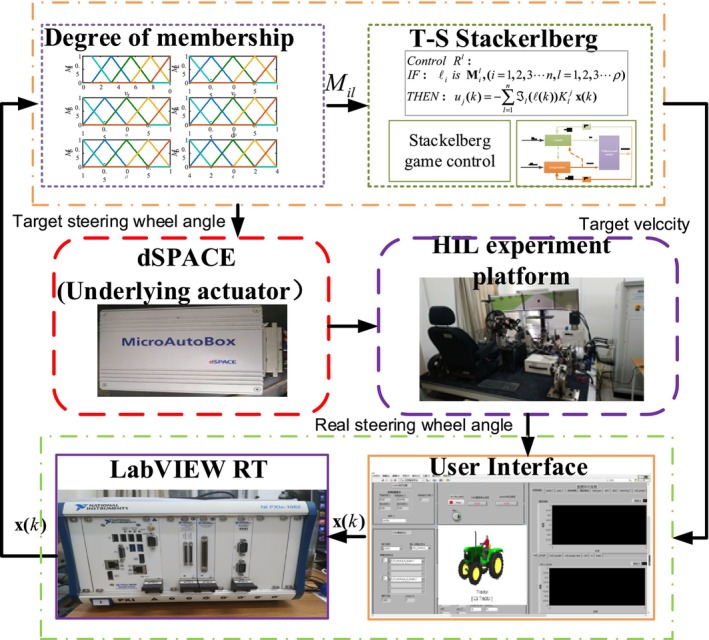
Diagram of the hardware‐in‐the‐loop experiment architecture.

To assess the efficacy of the lateral control and longitudinal control for intelligent agricultural machines, grounded in T‐S Stackelberg equilibrium theory, we established a hardware‐in‐the‐loop (HIL) experimental setup. As illustrated in Figure 4, CarSim software is integrated into the PXI system via LabVIEW to create a simulated environment. Meanwhile, the control algorithm developed in Simulink/Matlab is embedded within the dSPACE system through ControlDesk software. The overall HIL platform is constructed using dSPACE‐MicroAutoBox as the foundational driver, facilitating the execution of hardware‐in‐the‐loop experiments.

The fixed parameter values of the model established in the experiment are shown in Table 1.

**TABLE 1:** Parameter reference table.

Symbol	The variable name	Value
*m*	Total vehicle mass	1800
*I*_z_	Rotational inertia of the Z‐axis	2313
*l*_*f*_	Front axle wheelbase	1.89
*l*_*r*_	Rear axle wheelbase	1.61
*C*_*f*_	Equivalent turning stiffness of front axle	13,000
*C*_*r*_	Equivalent turning stiffness of rear axle	16,000

**FIGURE 5:**
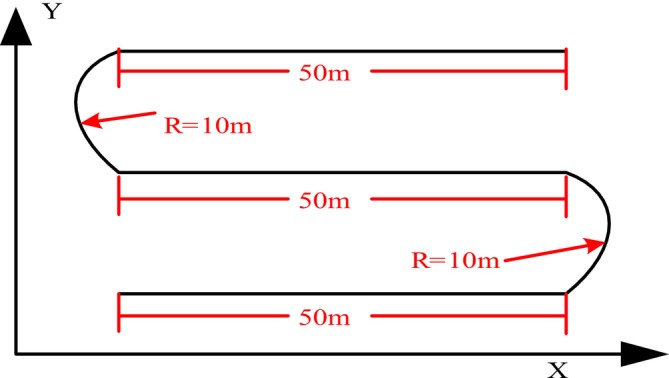
Target path setting diagram.

Figure 5 presents the configuration diagram of the target path. In this study, the configuration is designed as alternating sections of straight lines and curves to approximate the actual route of agricultural machinery field operations.

**FIGURE 6:**
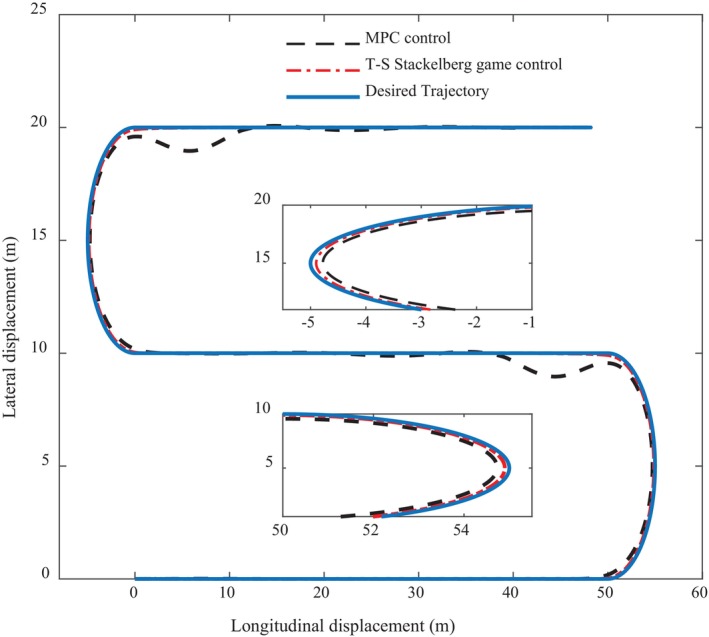
Path tracking control trajectory contrast curve.

Figure 6 shows the curve comparison diagram of intelligent driving agricultural machinery in the process of longitudinal and lateral path following control, and the MPC control algorithm is used for comparison in the experiment to verify the practicability and reliability of T‐S Stackelberg game control. From the overall trend in the figure, it can be clearly seen that the path following curve under MPC control has obvious errors; especially after two turns, there is bound to be a fluctuation, which is also the limitation of the MPC algorithm in the process of balancing multi‐agent control and reflects the advantages of T‐S Stackelberg game control. This is of great significance for agricultural machinery. When working in the field, the accuracy of control ensures the efficiency and quality of agricultural machinery.

**FIGURE 7:**
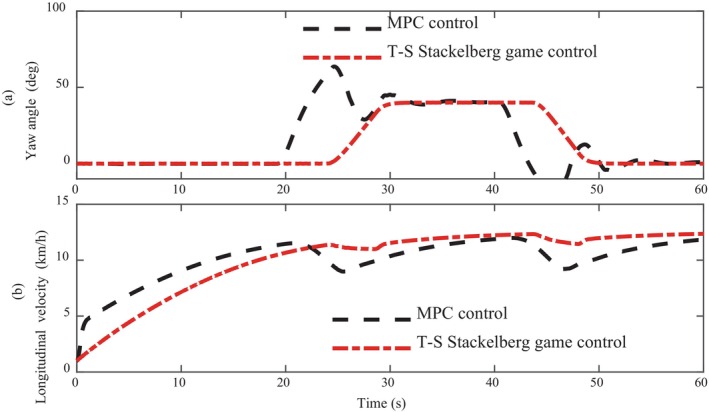
Comparison of yaw angle and longitudinal velocity.

Figure 7 shows the yaw Angle trajectory curve and longitudinal speed curve of intelligent driving agricultural machinery in geodetic coordinates. From the yaw Angle curve, it can be clearly seen that the intelligent agricultural machinery under MPC control has a large amplitude of vibration at 20s and 46 s, which indicates that the intelligent driving agricultural machinery has deviated from the target path, and this vibration can be clearly seen in the body. This is also a very poor experience for automatic operations, and on the contrary, the excellent stability of the proposed algorithm is set off. In addition, the rise time of yaw Angle under MPC control has already started in the 20s, which is 5 s earlier than that under T‐S Stackelberg game control, which indicates that the vehicle speed under MPC control is relatively fast, as can be clearly seen from the speed in Fig. (b). However, in terms of speed stability control, the T‐S Stackelberg game control is more stable, which indicates that the accuracy of its longitudinal control is also high.

**FIGURE 8:**
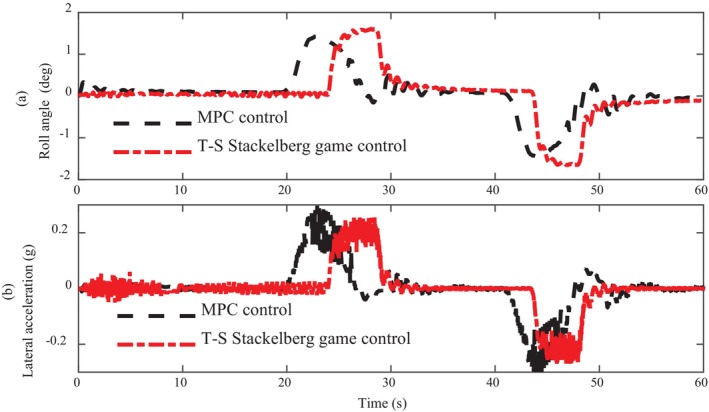
Comparison of roll angle and lateral acceleration.

Figure 8 shows the comparison curve of roll Angle and lateral acceleration of intelligent driving agricultural machinery. From the overall trend, there is little difference in the amplitude of roll Angle between the two algorithms, but from the details, the roll Angle under MPC control has many oscillations around the 30s and 45 s. The inclination Angle controlled by T‐S Stackelberg game is alleviated and has no fluctuation, which shows that the proposed algorithm has significant advantages in terms of comfort. The side Angle is a sign of stability and comfort; once the side Angle shocks, it is difficult to ensure the accuracy of agricultural machinery in the process of operation. The operator's sense of experience will become extremely poor, and even serious fear, which is very unfavorable to the development of intelligent agricultural machinery.

**FIGURE 9:**
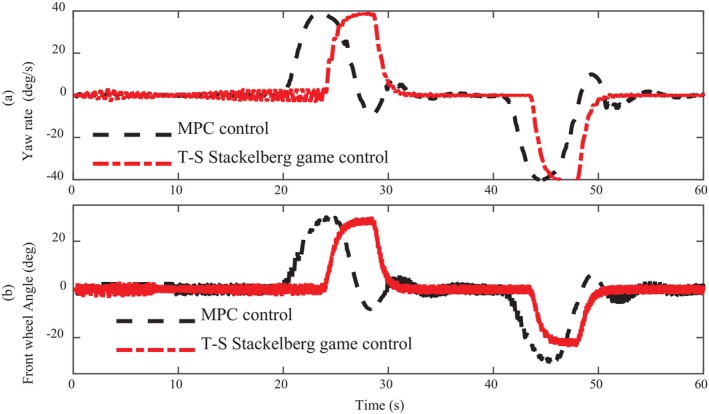
Comparison of yaw rate and front wheel angle.

Figure 9 shows the curve comparison between yaw velocity and front wheel angle of the two algorithms. On the whole, the yaw velocity curve controlled by the T‐S Stackelberg algorithm is smoother, while the yaw velocity controlled by the MPC algorithm fluctuates twice before and after 30 and 50 s. The cause can be found in the front wheel angle curve. Under the control of the MPC algorithm, the front wheel angle swings at 30 and 50 s, which is the direct cause of yaw velocity fluctuation. Analyzing the control input of these two algorithms, the reasons can be found from two aspects, which are path tracking error and speed control.

**FIGURE 10:**
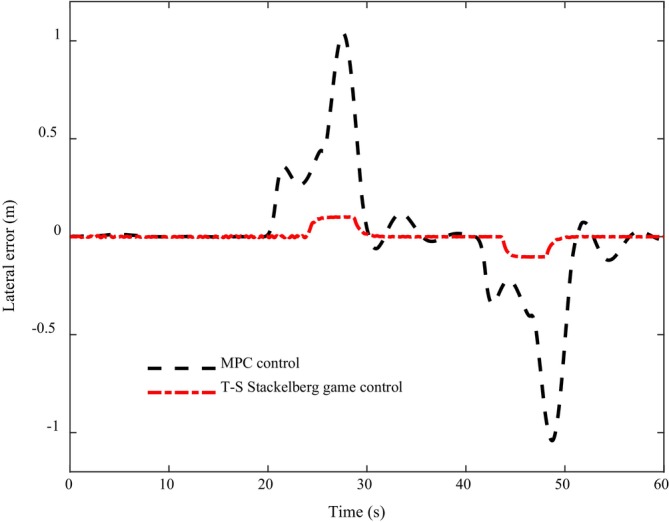
Comparison plot of tracking path error.

Figure 10 shows the lateral error comparison curve of the two algorithms in the process of path tracking. It can be intuitively seen from the figure that the error amplitude under the control of the T‐S Stackelberg algorithm is lower than 0.1 m, while the path tracking error under the control of the MPC algorithm is larger, especially at the 28th and 50th seconds, where the error is the largest, having exceeded 1 m. Moreover, the error lasts for a long time, and the error appears in the time periods of 20–35 and 40–55 s. Both from the perspective of the amplitude of the error and the duration of the error, the T‐S Stackelberg algorithm performs well. From the perspective of control, the T‐S Stackelberg algorithm considers the information interaction between multi‐objective and multi‐system, and solves the influence of model parameter change on controller performance, so that it performs better in the face of a complex environment in the horizontal and vertical control of intelligent driving.

## 6 Conclusion

This paper proposed a T‐S Stackelberg game control algorithm to address the coupling interference problem in intelligent agricultural machinery control and the performance deterioration caused by real‐time parameter variations. Through systematic design of the T‐S fuzzy model and Stackelberg game controller, we achieved superior tracking performance and stable speed control in HIL experiments compared to conventional methods.

### 6.1 Limitations and Future Research Directions

1. Stackelberg Game Implications

The current hierarchical leader‐follower structure assumes perfect information exchange, which may not hold in practical field conditions with communication delays. The unilateral decision‐making paradigm may need adaptation for scenarios requiring more cooperative steering strategies.

2 T‐S Fuzzy Model Constraints

The model's performance depends heavily on the pre‐defined membership functions, which were designed for typical operating conditions. Extreme environmental variations may challenge the model's interpolation capabilities.

3 Future Research Priorities

Implementation of cooperative game frameworks for scenarios where strict hierarchy is suboptimal. Real‐world validation under diverse field conditions to test robustness against unmodeled disturbances. Integration with multi‐agent coordination for fleet operations of agricultural machinery. Development of adaptive membership function mechanisms for broader operating envelopes.

These extensions will be investigated in our upcoming real‐vehicle tests and field operational evaluations, with particular focus on bridging the gap between theoretical control design and practical agricultural applications.

## Author Contributions

**Guangfei Xu:** writing – original draft (equal). **Dexi Wu:** data curation (equal), validation (equal). **Haizhu Xu:** investigation (equal), methodology (equal), resources (equal). **Hequan Miao:** software (equal), validation (equal). **Yulong Chen:** software (equal), writing – review and editing (equal).

## Conflicts of Interest

The authors declare no conflicts of interest.

## Data Availability

The data that support the findings of this study are not publicly available due to containing commercially sensitive information. Data are however available from the corresponding author upon reasonable request and with permission from the relevant ethics committee or institutional review board.

## Appendix A

**Table:**

Symbol	The variable name
*m*	Total vehicle mass
*I*_z_	Rotational inertia of the Z‐axis
*l*_*f*_	Front axle wheelbase
*l*_*r*_	Rear axle wheelbase
*l*_*w*_	Wheel base
$C_{\mathrm{\alpha f}}$	Equivalent turning stiffness of front axle
$C_{\mathrm{\alpha r}}$	Equivalent turning stiffness of rear axle
*v*_*x*_	Longitudinal velocity
*v*_*y*_	Lateral velocity
ψ	Yaw angle
$\dot{\psi }$	Yaw rate
X	Longitudinal displacement
Y	Lateral displacement
$F_{\mathrm{cf}}$	Front wheel lateral force
$F_{cr}$	Rear wheel lateral force
$u_{\delta }$	Front wheel angle
$u_{a\text{ccel}}$	Acceleration input
$\alpha_{f}$	Front wheel camber
$\alpha_{r}$	Rear wheel camber
$\delta_{f}$	Steering wheel angle
β	Centroid deflection angle
$\ell \left(k\right)$	Antecedent variable
$R^{l}$	Plant rule
$M_{i}^{l}$	Fuzzy set
$\mu_{l}\left(\ell \left(k\right)\right)$	The membership degree of the antecedent variable
